# TLR4/MyD88 expression patterns and novel genetic variants: association with aggressive clinicopathological features in colorectal cancer

**DOI:** 10.3389/fonc.2025.1568729

**Published:** 2025-07-09

**Authors:** Thai Tra Dang, Viet Nhat Pham, Ngoc Dung Tran, Thu Hang Ngo, Van Mao Can, Huy Hoang Nguyen, Thi Xuan Nguyen, Thanh Chung Dang

**Affiliations:** ^1^ Department of Pathology and Forensic Medicine, Military Hospital 103, Vietnam Military Medical University, Hanoi, Vietnam; ^2^ Department of Pathophysiology, Vietnam Military Medical University, Hanoi, Vietnam; ^3^ Institute of Biology, Vietnam Academy of Science and Technology, Hanoi, Vietnam

**Keywords:** TLR4/MyD88 pathway, colorectal carcinogenesis, genetic polymorphisms, molecular biomarkers, clinicopathological features, microsatellite instability

## Abstract

**Background:**

Toll-like receptor 4 (TLR4) and myeloid differentiation factor 88 (MyD88) signaling play a critical role in colorectal cancer (CRC) development. Despite extensive research, the relationship between genetic variations and protein expression patterns during adenoma-carcinoma progression remains poorly understood.

**Methods:**

We conducted a cross-sectional study of 176 CRC patients and 131 adenoma patients. Inclusion criteria required histologically confirmed primary colorectal tumors with adequate tissue content (≥30% tumor cells). TLR4 and MyD88 protein expression was evaluated using immunohistochemistry with standardized scoring systems. DNA sequencing identified genetic variants in TLR4 and MyD88 genes. Multivariate analyses assessed associations between protein expression, genetic variants, and clinicopathological features.

**Results:**

TLR4 expression was significantly higher in CRC compared to adenomas (66.5% vs 30.5%, p<0.001), with MyD88 showing widespread expression in both groups (CRC: 97.2%, adenoma: 95.4%). We identified novel variants in TLR4 (9:117713042) and MyD88 (rs138284536), significantly associated with increased CRC risk (OR=8.92, 95% CI: 1.14-69.95, p=0.037 and OR=20.01, 95% CI: 4.72-84.83, p<0.001, respectively). The MyD88 variant correlated with aggressive features including mucinous histology (43.5% vs 22.7%, p=0.036), advanced pT stage (29.6% vs 13.2%, p=0.044), and perineural invasion (61.5% vs 22.1%, p=0.004). Combined TLR4/MyD88 scores ≥5 significantly predicted lymph node metastasis (42.9% vs 28.3%, p=0.046) and high-grade tumor budding (p=0.002).

**Conclusions:**

Our study identifies distinct TLR4/MyD88 expression patterns in CRC progression and novel genetic variants associated with aggressive tumor features. These molecular alterations may serve as potential biomarkers for risk stratification and prognostic assessment in CRC patients, while offering promising targets for therapeutic intervention.

## 1 Introduction

Colorectal cancer (CRC) remains a significant global health burden, ranking third in incidence and second in mortality among all cancers worldwide (1). According to GLOBOCAN 2022, over 1.9 million new CRC cases and 900,000 deaths were reported globally (1). By 2030, the global burden of CRC is projected to increase by 60%, with more than 2.2 million new cases and 1.1 million deaths annually (2), particularly affecting transitioning countries due to lifestyle changes and aging populations (3).

Growing evidence suggests that chronic inflammation plays a crucial role in colorectal carcinogenesis (4, 5). Inflammatory cells create a tumor-promoting microenvironment by secreting cytokines and growth factors that enhance neoplastic cell survival, promote angiogenesis, and facilitate metastasis (4). These inflammatory mediators activate NFκB-dependent signaling pathways and regulate the expression of cancer-related genes, while oxidative stress associated with chronic inflammation can promote mutations favorable for tumor development (5, 6). The link between inflammation and cancer is particularly relevant in the colon, where continuous exposure to microbial products can modulate inflammatory responses (7).

The Toll-like receptor (TLR) family, particularly TLR4, has emerged as a key player in linking inflammation to cancer development (8, 9). TLR4 is expressed not only on immune cells but also on intestinal epithelial and tumor cells, mediating complex interactions within the tumor microenvironment (10). TLR4 signaling operates through both myeloid differentiation factor 88 (MyD88)-dependent and independent pathways, potentially promoting tumor progression by enhancing tumor cell adhesion, invasion, and metastasis via NF-κB regulation (10, 11). Furthermore, innate immune responses to gut microbiota through TLR4 signaling have been implicated in gastrointestinal malignancies (12), with experimental evidence showing that blocking TLR4-MyD88 signaling suppresses inflammation-associated tumor development (11, 13).

Recent studies revealed complex TLR4/MyD88 signaling interactions with tumor progression (14, 15). However, critical knowledge gaps persist. First, coordinated TLR4/MyD88 expression during adenoma-carcinoma progression remains poorly characterized (16). Second, the combined impact of genetic variants on protein expression and clinicopathological features requires clarification (17, 18). Third, pathway relationships with molecular CRC subtypes, particularly MSI status and tumor-infiltrating lymphocytes, need investigation (19, 20).

This study aims to address these gaps by: (1) analyzing the immunohistochemical expression patterns of TLR4 and MyD88 proteins in colorectal adenomas and carcinomas, (2) investigating single nucleotide polymorphisms (SNPs) in TLR4 and MyD88 genes, and (3) evaluating their associations with clinicopathological features and protein expression. Our findings could provide insights into the role of TLR4/MyD88 signaling in colorectal carcinogenesis and identify potential therapeutic targets.

## 2 Materials and methods

### 2.1 Study design and population

This cross-sectional study with retrospective and prospective components was conducted at Military Hospital 103, Vietnam Military Medical University, between April 2021 and October 2023. Sample size was calculated using the formula for comparing two proportions:


$$n\;=Z^{2}\left[p\left(1-p\right)\right]/d^{2}$$

where Z = 1.96 (95% confidence level), d = 0.05 (margin of error), and p represents the expected variant frequency based on previous studies (15, 17). The calculation indicated a minimum required sample size of 133 cases for adequate statistical power. To account for potential technical failures and missing data, we enrolled 176 CRC patients and 131 controls.

Retrospective data were collected from surgical specimens and medical records from April 2021 to December 2022, while prospective enrollment was conducted from January 2023 to October 2023 using consecutive sampling. The study protocol was approved by the Ethics Committee of Military Hospital 103 (Decision No. 06/CNChT-HĐĐĐ, dated January 6, 2023) and was performed in accordance with the Declaration of Helsinki. Written informed consent was obtained from all participants or their legal representatives prior to enrollment.

Based on the WHO classification of colorectal tumors (21) and European Society for Medical Oncology (ESMO) guidelines (22), patients with histologically confirmed primary colorectal adenocarcinoma who underwent surgical resection were included in the CRC group. Inclusion criteria required patients to be aged ≥18 years with available formalin-fixed paraffin-embedded tissue blocks containing adequate tumor content (≥30% tumor cells) and complete clinical and pathological data. The control group comprised patients with histologically confirmed colorectal adenomas through colonoscopic biopsy, classified according to WHO criteria (21). Exclusion criteria for both groups encompassed: previous history of any malignancy, preoperative chemotherapy or radiotherapy, inflammatory bowel disease, hereditary colorectal cancer syndromes, synchronous or metachronous tumors, inadequate tissue samples, or incomplete clinical data.

### 2.2 Clinical and pathological assessment

Clinical data were systematically collected from medical records following a standardized protocol. Tumor location was classified according to anatomical landmarks: right colon (from cecum to proximal two-thirds of transverse colon), left colon (from distal third of transverse colon to sigmoid colon), and rectum.

All surgical specimens were processed according to standard pathology procedures. Pathological staging was performed according to the 8th edition of the American Joint Committee on Cancer (AJCC) TNM classification system (23). Two independent pathologists reviewed all H&E-stained slides. For CRC cases, histopathological evaluation included assessment of histological type (conventional adenocarcinoma, mucinous adenocarcinoma, or other variants including signet ring cell carcinoma and poorly differentiated carcinoma), tumor differentiation grade (well, moderate, or poor), depth of invasion (pT stage), lymphovascular invasion, perineural invasion, and lymph node status. Tumor differentiation grading was applied only to conventional adenocarcinomas following WHO criteria, which define well-differentiated (>95% glandular component), moderately differentiated (50-95% glandular component), and poorly differentiated (<50% glandular component) categories (21). Mucinous adenocarcinomas (n=25) were excluded from differentiation grading as WHO classification does not apply conventional grading criteria to this histological variant. MSI status testing (n=151) was performed based on clinical indications according to standard practice guidelines for CRC patients.

Tumor budding was assessed according to International Tumor Budding Consensus Conference criteria (24). This involved counting tumor buds, defined as single tumor cell or cluster of up to 4 cells, in hotspot areas (0.785 mm²) at 20× magnification. Cases were categorized as low (0–4 buds), intermediate (5–9 buds), or high (≥10 buds) grade. Tumor-infiltrating lymphocytes (TILs) were evaluated following International TILs Working Group recommendations (25), focusing on stromal TILs in both central tumor and invasive margin regions.

For adenoma specimens, pathological assessment included evaluation of adenoma type (conventional, sessile serrated, or traditional serrated), grade of dysplasia, size, and configuration. All pathological assessments were performed blinded to clinical information and molecular analysis results. Inter-observer variability was assessed using Cohen’s kappa coefficient, with discordant cases resolved through consensus review.

### 2.3 Immunohistochemistry

Immunohistochemical staining was performed on 4-μm sections from formalin-fixed, paraffin-embedded tissue blocks. Six monoclonal antibodies were used: anti-PMS2 (EP51), anti-MLH1 (M1), anti-MSH2 (G219-1129), anti-MSH6 (SPO3) (all from Leica, Germany), and anti-TLR4 (HTA125: sc-13593) and anti-MyD88 (B-1: sc-136970) (both from Santa Cruz Biotech, Santa Cruz, CA, USA). After deparaffinization in xylene and rehydration through graded ethanol, antigen retrieval was performed using pressure cooking in citrate buffer (pH 6.0). Endogenous peroxidase activity was blocked with 0.3% hydrogen peroxide for 30 minutes.

The sections were incubated with primary antibodies overnight at 4°C following manufacturer’s recommendations. The streptavidin-biotin-peroxidase complex technique was employed using biotinylated secondary antibody and streptavidin-labeled peroxidase (DakoCytomation, Glostrup, Denmark) with 50-minute incubation at room temperature. Immunoreactivity was visualized using 3,3’-diaminobenzidine as chromogen, with 3-amino-9-ethylcarbazole reaction for confirmation. Sections were counterstained with hematoxylin. Human tonsil tissue served as positive control for TLR4, MyD88, and MMR proteins expression. Phosphate-buffered solution without primary antibody was used as negative control.

For immunohistochemical evaluation, two independent pathologists assessed all slides without knowledge of clinical data, following the method described by Wang et al. (8). Any membranous and/or cytoplasmic staining was considered positive for TLR4 and MyD88. Digital images of representative high-power fields (200×) were captured using a Leica microscope-mounted digital camera. An average of 2,000 tumor cells per case were evaluated. Results were expressed as percentage of positive tumor cells and scored on a scale of 0-4 (0: no staining; 1+: <10%; 2+: 11-30%; 3+: 31-50%; 4+: >50%). Expression levels were then categorized as negative (0), low (1+ and 2+), or high (3+ and 4+). Combined TLR4/MyD88 scores were calculated as the simple arithmetic sum of individual ordinal expression scores (TLR4: 0–4 scale + MyD88: 0–4 scale, maximum possible combined score = 8), then dichotomized as <5 or ≥5 following the literature-based approach described by Wang et al. (8). For MMR proteins, nuclear staining was evaluated according to established criteria (26), with cases showing complete loss of expression in tumor cells with preserved internal control staining considered MMR-deficient.

### 2.4 DNA sequencing and variant analysis

Genomic DNA was isolated from formalin-fixed, paraffin-embedded tissue samples using the GeneJET FFPE DNA Extraction Kit (Thermo Scientific, Waltham, MA, USA). PCR amplification was performed using the following primers: TLR4-F: 5’-AGTTTGACAAATCTGCTCTAG-3’ and TLR4-R: 5’-TGGTAATAACACCATTGAAGCTCAG-3’; MyD88-F: 5’-AACCCTGGGGTTGAAGACTG-3’ and MyD88-R: 5’-GGCGAGTCCAGAACCAAGAT-3’. The expected amplicon lengths were 429 bp for TLR4 and 207 bp for MyD88. PCR products were purified using the GeneJET PCR Purification Kit (Thermo Scientific).

DNA sequencing was performed using the 3500 Genetic Analyzer (Thermo Scientific). GenBank accession numbers NM_138554.5 and NM_002468.5 were used as reference sequences for TLR4 and MyD88, respectively. Sequencing was performed bidirectionally using the same primers as for PCR amplification. Sequence variants were confirmed by repeated PCR amplification and sequencing of both DNA strands. The potential functional impact of identified variants was predicted using PolyPhen-2 (27), with multiple sequence alignment performed to evaluate evolutionary conservation. All sequence analyses were performed by two independent investigators blinded to clinical data, with discrepancies resolved through consensus review.

### 2.5 Statistical analysis

Statistical analyses were performed using SPSS version 20 (IBM, New York, NY, USA) and GraphPad Prism version 8.4 (GraphPad Software, San Diego, CA, USA). Demographic and clinical characteristics were compared between groups using Chi-square test or Fisher’s exact test for categorical variables, and Student’s t-test or Mann-Whitney U test for continuous variables as appropriate. For immunohistochemical scoring analysis, inter-observer agreement was assessed using Cohen’s kappa coefficient.

Correlations between TLR4 and MyD88 expression were evaluated using Pearson’s and Spearman’s correlation coefficients. For genetic variant analysis, Hardy-Weinberg equilibrium was tested using chi-square goodness-of-fit test in the control group. Logistic regression was used to calculate odds ratios (ORs) and 95% confidence intervals (CIs) for genetic associations, with adjustment for age and gender. Multiple testing corrections were applied using the Bonferroni method for primary analyses and False Discovery Rate (FDR) for exploratory analyses. Gene-gene interaction analysis was performed using likelihood ratio tests.

Statistical significance was set at p < 0.05 (two-sided) for all tests, with adjusted p-values reported for multiple comparisons.

## 3 Results

### 3.1 Clinical and pathological characteristics

The study included 176 colorectal cancer (CRC) patients and 131 colorectal adenoma patients with complete clinical and pathological data (**Table 1**). Analysis of demographic features revealed that CRC patients were significantly older than adenoma patients (mean age ± SD: 66.03 ± 13.43 vs 62.44 ± 13.53 years, p=0.021), while gender distribution was comparable between groups (male: 62.5% vs 61.8%, p=0.892).

**Table 1:** Clinical and pathological characteristics of the study population.

Characteristics	CRC (N=176)	Adenoma (N=131)	*P*-value
Demographic Features			
Age (years)†	66.03 ± 13.43	62.44 ± 13.53	0.021*
Sex‡			
Male	110 (62.5)	81 (61.8)	0.892
Female	66 (37.5)	50 (38.2)	
Tumor Characteristics			
Location‡			
Right colon	57 (32.4)	31 (23.7)	0.002*
Left colon	88 (50.0)	54 (41.2)	
Rectum	31 (17.6)	46 (35.1)	
Size			
Mean (cm)†	4.47 ± 1.90	1.43 ± 0.86	<0.001*
≥5 cm‡	69 (39.2)	1 (0.8)	<0.001*
CRC Pathological Features	n	%	
Histological type			
Conventional adenocarcinoma	149	84.7	
Mucinous adenocarcinoma	25	14.2	
Others§	2	1.1	
Differentiation (n=151)¥			
Well/Moderate	142	94.0	
Poor	9	6.0	
Tumor staging			
pT1-2	43	24.5	
pT3-4	133	75.5	
Lymph node metastasis	70	39.8	
Distant metastasis	14	8.0	
Vascular invasion	18	10.2	
Perineural invasion	13	7.4	
Tumor-infiltrating lymphocytes			
High/Moderate	52	29.5	
Low	124	70.5	
MSI status (n=151)¶			
MSS	106	70.2	
MSI-H	45	29.8	

†Mean ± standard deviation, analyzed using Student’s t-test; ‡Number (percentage), analyzed using Student’s t-test; §Including signet ring cell carcinoma and poorly differentiated carcinoma; ¥Differentiation grading applies only to conventional adenocarcinomas per WHO criteria; ¶MSI testing performed based on clinical indications; *Statistically significant (p<0.05) CRC, Colorectal cancer; MSI, Microsatellite instability; MSS, Microsatellite stable; MSI-H, High-level microsatellite instability.

Anatomical distribution showed distinct patterns between CRC and adenoma groups (p=0.002). CRC lesions predominantly affected the left colon (50.0%), followed by right colon (32.4%) and rectum (17.6%). In contrast, adenomas demonstrated more even distribution between left colon (41.2%) and rectum (35.1%). CRC tumors exhibited significantly larger size compared to adenomas (mean diameter ± SD: 4.47 ± 1.90 vs 1.43 ± 0.86 cm, p<0.001), with 39.2% of CRC tumors measuring ≥5 cm versus only 0.8% of adenomas.

Detailed pathological assessment of CRC cases revealed conventional adenocarcinoma as the predominant histological type (84.7%), followed by mucinous adenocarcinoma (14.2%). Most tumors demonstrated well or moderate differentiation (94.0%). Advanced disease characteristics included pT3–4 stage (75.5%), lymph node metastases (39.8%), and distant metastases (8.0%). Vascular and perineural invasion were identified in 10.2% and 7.4% of cases, respectively. Analysis of tumor microenvironment features showed low tumor-infiltrating lymphocytes in 70.5% of cases. Among the 151 cases evaluated for microsatellite instability status, 29.8% exhibited MSI-H phenotype.

### 3.2 TLR4 and MyD88 expression patterns

#### 3.2.1 Expression patterns in CRC and adenoma tissues

**Figure 1:**
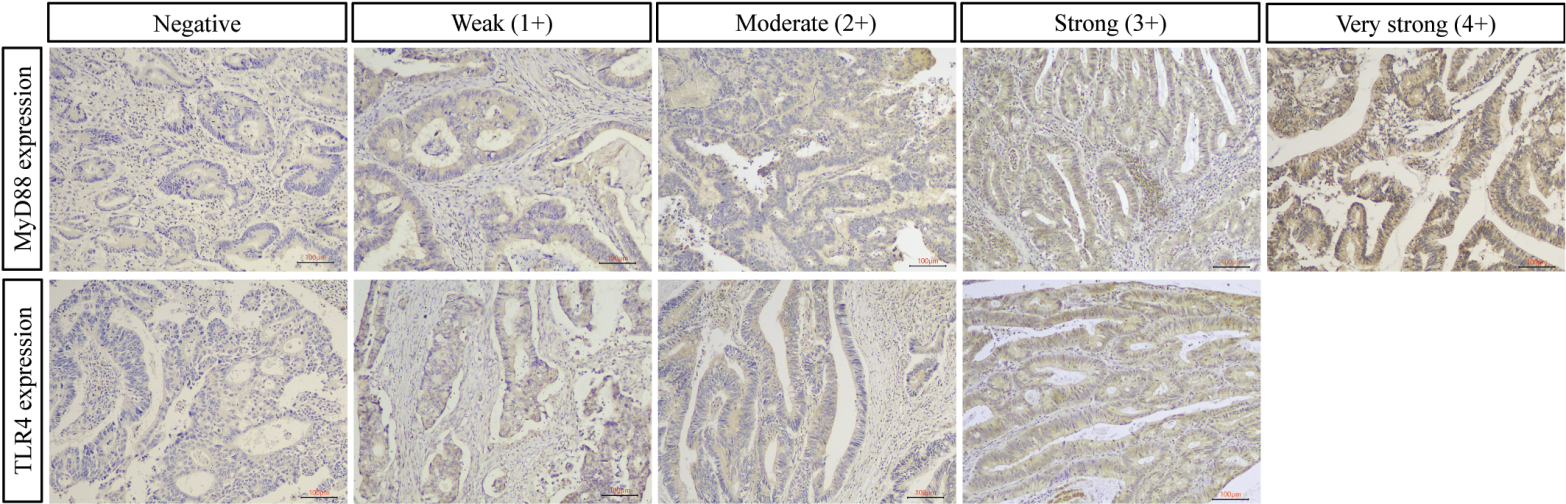
Representative immunohistochemical staining of TLR4 and MyD88 in colorectal cancer. Representative images showing protein expression scoring patterns. Upper panel: MyD88 immunostaining ranging from negative (0) to very strong (4+). Lower panel: TLR4 immunostaining ranging from negative (0) to strong (3+). MyD88 shows cytoplasmic staining pattern, while TLR4 exhibits both membranous and cytoplasmic staining. Expression intensity was scored on standardized scales (0-4+ for MyD88; 0-3+ for TLR4) by two independent pathologists. For quantitative analysis, scores were categorized as low (0, 1+, 2+) or high (3+/4+ for MyD88; 3+ for TLR4). Original magnification ×100, scale bar = 100 μm.

Immunohistochemical analysis revealed distinct expression patterns of TLR4 and MyD88 proteins in colorectal tissues (**Figure 1**). TLR4 expression was significantly higher in CRC compared to adenoma cases (66.5% vs 30.5%, p<0.001). Among CRC cases, 5.1% showed strong (3+) TLR4 expression, while 61.4% exhibited low expression (1+/2+). In adenomas, TLR4 expression was exclusively of low intensity when present.

MyD88 demonstrated widespread expression in both groups with comparable overall positivity rates (CRC: 97.2%, adenoma: 95.4%, p=0.518). In CRC cases, high MyD88 expression (3+/4+) was predominant (58.5%), comprising very strong (4+, n=54) and strong (3+, n=49) staining patterns. Low expression (1+/2+) was observed in 38.7% of cases. The distribution of expression patterns is detailed in **Table 2**.

**Table 2:** Immunohistochemical expression analysis of TLR4 and MyD88: comparison between CRC and adenoma cases.

Expression Level	CRC (n=176)	Adenoma (n=131)	*P*-value
TLR4 expression			
Negative	59 (33.5)	91 (69.5)	<0.001*
Low (1+/2+)	108 (61.4)	40 (30.5)	
High (3+)	9 (5.1)	0 (0)	
MyD88 expression			
Negative	5 (2.8)	6 (4.6)	0.518
Low (1+/2+)	68 (38.7)	44 (33.6)	
High (3+/4+)	103 (58.5)	81 (61.8)	
Combined score			
<5	116 (65.9)	115 (87.8)	<0.001*
≥5	60 (34.1)	16 (12.2)	

Values are presented as n (%); Statistical analysis was performed using Fisher’s exact test; CRC, Colorectal cancer; *Statistically significant (p<0.05).

#### 3.2.2 Co-expression analysis

**Figure 2:**
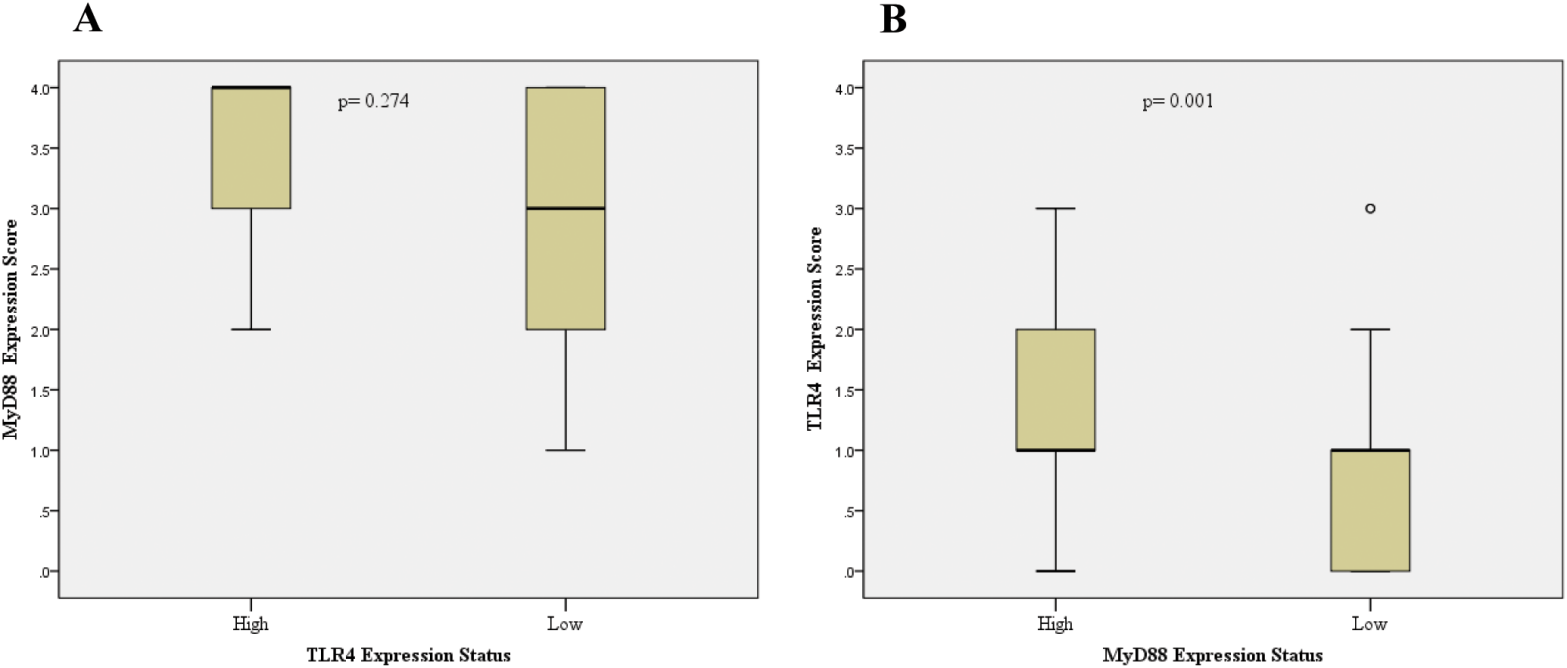
Analysis of TLR4 and MyD88 co-expression patterns in colorectal cancer. Box plots showing the relationship between TLR4 and MyD88 expression levels. **(A)** MyD88 expression scores in tumors with low versus high TLR4 expression (p=0.274). **(B)** TLR4 expression scores in tumors with low versus high MyD88 expression (p=0.001). Expression levels were dichotomized as low (0, 1+, 2+) or high (3+/4+ for MyD88; 3+ for TLR4). Boxes represent interquartile range with median line; whiskers show range; circles indicate outliers. Statistical analysis was performed using Mann-Whitney U test.

Combined TLR4/MyD88 expression analysis revealed significantly elevated scores (≥5) in CRC compared to adenoma cases (34.1% vs 12.2%, p<0.001). Correlation analysis demonstrated significant positive associations between TLR4 and MyD88 expression levels in CRC tissues (Pearson r=0.372, p<0.001; Spearman r=0.373, p<0.001). CRC cases with high MyD88 expression showed significantly higher TLR4 expression levels (p=0.001), although the inverse relationship was not statistically significant (p=0.274; **Figure 2**).

#### 3.2.3 Clinicopathological correlations

Protein expression patterns showed several significant associations with clinicopathological features (**Table 3**). High TLR4 expression significantly correlated with perineural invasion (30.0% vs 5.6%, p=0.029), with trends toward association with vascular invasion (17.6% vs 6.0%, p=0.123) and high-grade tumor budding (18.8% vs 5.4%, p=0.161).

**Table 3:** Analysis of TLR4 and MyD88 expression in relation to major clinicopathological parameters in CRC (N=176).

Parameters	TLR4 Expression			MyD88 Expression			Combined Score		
	Low	High	*P-value*	Low	High	*P-value*	<5	≥5	*P-value*
Tumor Invasion									
pT stage									
pT1-2	21 (91.3)	2 (8.7)	0.561	16 (40.0)	24 (60.0)	0.972	33 (76.7)	10 (23.3)	0.085
pT3-4	87 (92.6)	7 (7.4)		52 (39.7)	79 (60.3)		83 (62.4)	50 (37.6)	
Perineural invasion									
Absent	101 (94.4)	6 (5.6)	0.029*	65 (41.1)	93 (58.9)	0.201	110 (67.5)	53 (32.5)	0.136
Present	7 (70.0)	3 (30.0)		3 (23.1)	10 (76.9)		6 (46.2)	7 (53.8)	
Vascular invasion									
Absent	94 (94.0)	6 (6.0)	0.123	63 (41.2)	90 (58.8)	0.272	107 (67.7)	51 (32.3)	0.133
Present	14 (82.4)	3 (17.6)		5 (27.8)	13 (72.2)		9 (50.0)	9 (50.0)	
Metastasis Spread									
Lymph node metastasis									
Absent	64 (91.4)	6 (8.6)	0.739	44 (43.1)	58 (56.9)	0.273	76 (71.7)	30 (28.3)	0.046*
Present	44 (93.6)	3 (6.4)		24 (34.8)	45 (65.2)		40 (57.1)	30 (42.9)	
Tumor Progression									
Tumor budding									
Low	70 (94.6)	4 (5.4)	0.161	55 (46.2)	64 (53.8)	0.022*	92 (74.2)	32 (25.8)	0.002*
Intermediate	25 (92.6)	2 (7.4)		10 (29.4)	24 (70.6)		16 (47.1)	18 (52.9)	
High	13 (81.3)	3 (18.8)		3 (16.7)	15 (83.3)		8 (44.4)	10 (55.6)	

Data presented as number (percentage); Statistical analysis was performed using the Chi-square test or Fisher’s exact test where appropriate; *Statistically significant (p<0.05; CRC, Colorectal cancer.

MyD88 expression demonstrated a significant association with tumor budding grade (p=0.022), showing progressive increases from low-grade (53.8%) through intermediate-grade (70.6%) to high-grade tumors (83.3%). Combined TLR4/MyD88 scores ≥5 correlated significantly with lymph node metastasis (42.9% vs 28.3%, p=0.046) and tumor budding grade (p=0.002). A trend was observed between combined scores ≥5 and advanced pT stage (37.6% in pT3–4 vs 23.3% in pT1-2, p=0.085)–. The association between MyD88 expression and tumor budding grade demonstrates biological plausibility, as MyD88-dependent signaling promotes epithelial-mesenchymal transition programs crucial for tumor invasion. The progressive increase in MyD88 expression from low-grade (53.8%) through intermediate-grade (70.6%) to high-grade (83.3%) tumor budding supports a dose-response relationship between pathway activation and invasive potential. Comprehensive analysis of protein expression in relation to all clinicopathological parameters, including age, tumor location, size, differentiation grade, and immune features, revealed no additional statistically significant associations (**Supplementary Table S1**).

### 3.3 Genetic analysis

#### 3.3.1 Identification and distribution of variants

**Figure 3:**
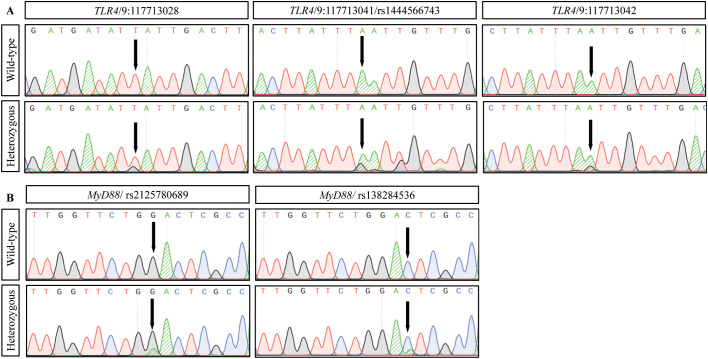
DNA sequence analysis of TLR4 and MyD88 variants in colorectal cancer. Representative DNA sequencing chromatograms showing identified variants. **(A)** TLR4 variants: wild-type (top) and heterozygous variant (bottom) sequences at positions 9:117713028 (T>G), 9:117713041/rs1444566743 (A>G), and 9:117713042 (A>G). **(B)** MyD88 variants: wild-type (top) and heterozygous variant (bottom) sequences at rs2125780689 (G>A) and rs138284536 (C>A). Arrows indicate variant positions. Base changes are shown in parentheses (reference>variant). Sequence analysis was performed using standard Sanger sequencing methods.

DNA sequencing analysis identified five missense variants: three in TLR4 (rs1444566743, 9:117713028, and 9:117713042) and two in MyD88 (rs2125780689 and rs138284536). All variants maintained Hardy-Weinberg equilibrium in both study groups (**Table 4**). The distribution of variants showed distinct patterns between CRC and control groups, with genotype frequencies detailed in **Table 4**. Sequence analysis demonstrated clear heterozygous variant patterns, as shown by the chromatograms in **Figure 3**. All identified variants were observed only in heterozygous state; no homozygous variant genotypes were detected in either study group. This absence of homozygous variants precluded meaningful recessive model analysis and limited statistical power for comprehensive genetic model testing.

**Table 4:** Genetic analysis of TLR4 and MyD88 variants in CRC.

Gene/Variant	Position	MAF		HWE (*p*-value)		Genotype Distribution		OR (95% CI)	*P*-value
		Adenoma	CRC	Adenoma	CRC	Adenoma	CRC		
TLR4									
rs1444566743	9:117713041	0.041	0.048	0.925	0.853	AA: 78 (91.8)	AA: 113 (90.4)	1.00	–
						AG: 7 (8.2)	AG: 12 (9.6)	1.18 (0.45-3.14)	0.735
9:117713028	9:117713028	0.018	0.032	0.986	0.934	TT: 82 (96.5)	TT: 117 (93.6)	1.00	–
						TG: 3 (3.5)	TG: 8 (6.4)	1.87 (0.48-7.26)	0.366
9:117713042	9:117713042	0.006	0.048	0.999	0.854	AA: 84 (98.8)	AA: 113 (90.4)	1.00	–
						AG: 1 (1.2)	AG: 12 (9.6)	8.92 (1.14-69.95)	0.037*
MyD88									
rs2125780689	3:38141253	0.071	0.010	0.703	0.993	GG: 102 (85.7)	GG: 150 (98.0)	1.00	–
						GA: 17 (14.3)	GA: 3 (2.0)	0.12 (0.03-0.42)	<0.001*
rs138284536	3:38141255	0.008	0.128	0.996	0.196	CC: 117 (98.3)	CC: 114 (74.5)	1.00	–
						CA: 2 (1.7)	CA: 39 (25.5)	20.01 (4.72-84.83)	<0.001*

MAF, Minor allele frequency; HWE, Hardy-Weinberg equilibrium; OR, Odds ratio; CI, Confidence interval; Adenoma group: n=85 for TLR4, n=119 for MyD88; Colorectal cancer (CRC) group: n=125 for TLR4, n=153 for MyD88; Genotype data presented as number (percentage); Statistical analysis performed using chi-square test/Fisher’s exact test for frequencies and logistic regression for OR calculation; HWE tested using chi-square goodness-of-fit test; *Statistically significant (p<0.05).

### 3.4 Risk assessment for CRC

Genetic association analysis revealed significant relationships between specific variants and CRC risk (**Table 4**). The TLR4 9:117713042 variant showed increased CRC risk, with AG genotype being more frequent in cases versus controls (9.6% vs 1.2%, OR=8.92, 95% CI: 1.14-69.95, p=0.037). MyD88 variants demonstrated opposing effects: rs2125780689 GA genotype showed a protective association (2.0% vs 14.3%, OR=0.12, 95% CI: 0.03-0.42, p<0.001), while rs138284536 CA genotype was associated with increased risk (25.5% vs 1.7%, OR=20.01, 95% CI: 4.72-84.83, p<0.001). Extended genetic model analysis, including dominant, recessive models and allele-based analysis, confirmed these associations and is detailed in **Supplementary Table S2**. However, the wide confidence intervals for these variants reflect limited sample size and low variant frequencies, indicating substantial uncertainty around effect estimates. While associations appear strong, replication in larger cohorts is essential for validation given the limited statistical precision.

### 3.5 *In silico* functional prediction

**Figure 4:**
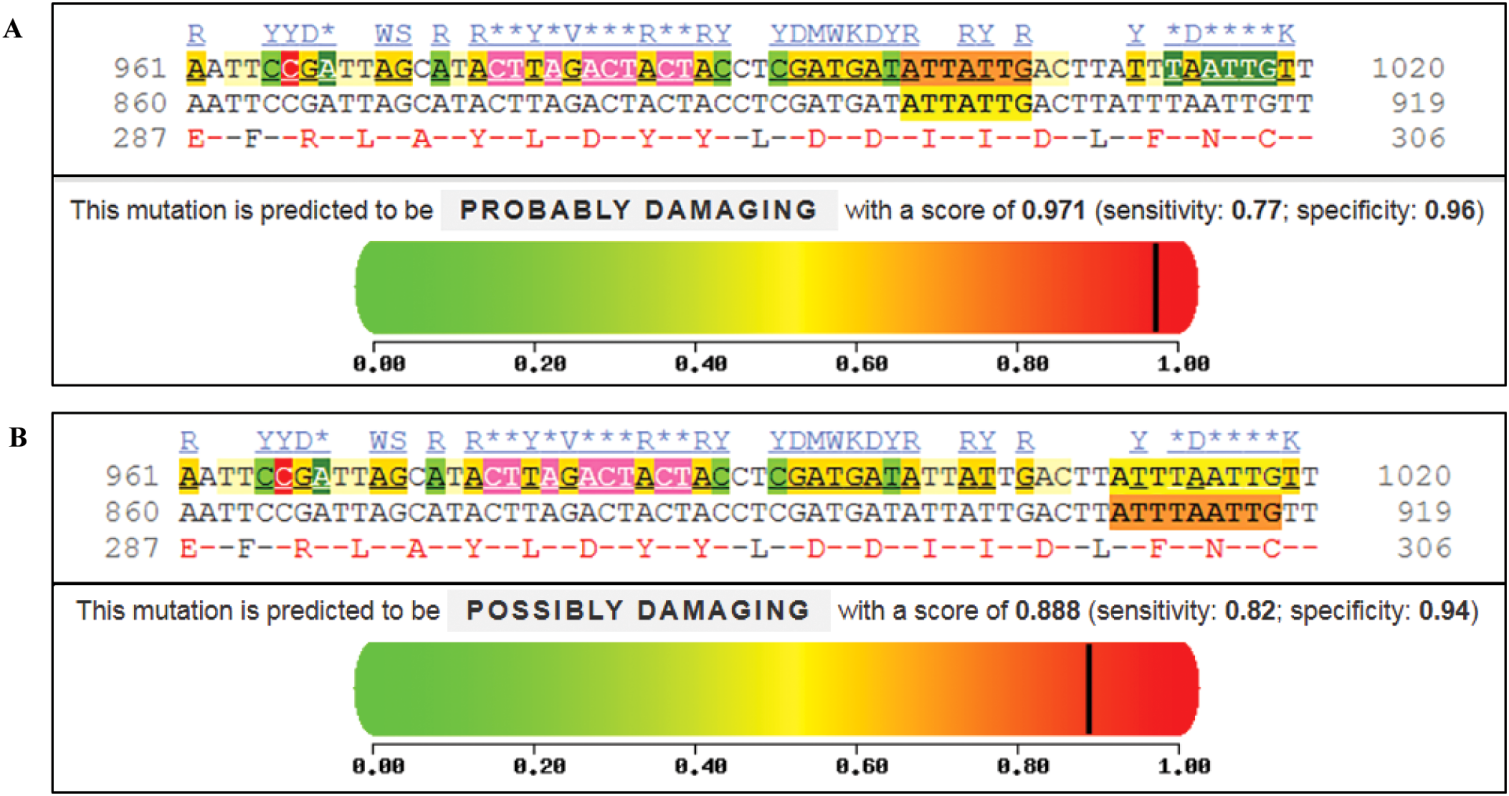
Functional impact analysis of TLR4 variants using PolyPhen-2. **(A)** Analysis of p.I300M variant (c.900A>G, chr9:117713028) showing “probably damaging” prediction (score: 0.971; sensitivity: 0.77; specificity: 0.96). **(B)** Analysis of p.N305S variant (c.914A>G, chr9:117713042) showing “possibly damaging” prediction (score: 0.888; sensitivity: 0.82; specificity: 0.94). For both variants, conservation analysis across species is shown below the prediction plots. Color scale represents predicted impact: green (benign, 0) to red (damaging, 1). Vertical black lines indicate variant-specific scores. Conserved amino acid positions are highlighted.

PolyPhen-2 analysis predicted potentially significant functional effects for both TLR4 variants (**Figure 4**). The p.I300M variant (9:117713028) was predicted to be probably damaging (score: 0.971, sensitivity: 0.77, specificity: 0.96). The p.N305S variant (9:117713042) was predicted to be possibly damaging (score: 0.888, sensitivity: 0.82, specificity: 0.94). Multiple sequence alignment demonstrated conservation of these positions across species. The predicted structural impacts, while computationally supported, must be interpreted cautiously given the absence of functional validation. The p.N305S substitution affects a conserved asparagine residue in the TLR4 extracellular domain, potentially altering ligand binding affinity, while the MyD88 variants may impact adapter protein interactions.

### 3.6 Genotype-phenotype associations and clinical correlations

Analysis of genotype-phenotype relationships revealed distinct patterns for TLR4 and MyD88 variants (**Table 5**). The TLR4 9:117713042 variant showed significant association with mucinous histology (23.5% vs 7.4% in conventional adenocarcinomas, p=0.038).

**Table 5:** Genotype-phenotype associations: analysis of genetic variants in relation to clinical and molecular features.

Features	TLR4 9:117713042			MyD88 rs138284536		
	Wild-ype	Variant	p	Wild-ype	Variant	p
Histological Features						
Histological type						
Conventional	99 (92.5)	8 (7.5)	0.038*	99 (77.3)	29 (22.7)	0.036*
Mucinous	13 (76.5)	4 (23.5)		13 (56.5)	10 (43.5)	
Disease Progression						
pT stage						
pT1-2	25 (78.1)	7 (21.9)	0.012*	33 (86.8)	5 (13.2)	0.044*
pT3-4	88 (94.6)	5 (5.4)		81 (70.4)	34 (29.6)	
Perineural invasion						
Absent	104 (91.2)	10 (8.8)	0.285	109 (77.9)	31 (22.1)	0.004*
Present	9 (81.8)	2 (18.2)		5 (38.5)	8 (61.5)	
Tumor Microenvironment						
TILs						
High/Moderate	35 (94.6)	2 (5.4)	0.507	42 (91.3)	4 (8.7)	0.002*
Low	78 (88.6)	10 (11.4)		72 (67.3)	35 (32.7)	
MSI status						
MSS	72 (90.0)	8 (10.0)	0.740	73 (78.5)	20 (21.5)	0.006*
MSI-H	28 (87.5)	4 (12.5)		22 (55.0)	18 (45.0)	
Protein Expression						
Expression level						
Low	75 (91.5)	9 (81.8)	0.288	38 (70.4)	16 (29.6)	0.404
High	7 (8.5)	2 (18.2)		72 (76.6)	22 (23.4)	
Combined score						
<5	67 (59.3)	5 (41.7)	0.240	67 (58.8)	29 (74.4)	0.082
≥5	46 (40.7)	7 (58.3)		47 (41.2)	10 (25.6)	

Data presented as number (percentage); Wild-type: AA for TLR4, CC for MyD88; Variant: AG for TLR4, CA for MyD88; Low expression: scores 0-2+; High expression: scores 3+ for TLR4, 3+/4+ for MyD88; Combined score represents the sum of TLR4 and MyD88 expression scores (Low: <5; High: ≥5); Statistical analysis performed using Chi-square test or Fisher’s exact test where appropriate. *Statistically significant (p<0.05) TILs, Tumor-infiltrating lymphocytes; MSI, Microsatellite instability; MSS, Microsatellite stable; MSI-H, High-level microsatellite instability.

MyD88 rs138284536 variant demonstrated multiple significant clinicopathological associations. It was more frequent in mucinous versus conventional adenocarcinomas (43.5% vs 22.7%, p=0.036), advanced pT stage (29.6% vs 13.2%, p=0.044), and cases with perineural invasion (61.5% vs 22.1%, p=0.004). This variant also showed significant associations with tumor microenvironment features, occurring more frequently in tumors with low TILs (32.7% vs 8.7%, p=0.005) and MSI-H status (45.0% vs 21.5%, p=0.006). This paradoxical relationship suggests that variant-mediated pathway alterations may influence both DNA repair mechanisms and immune cell recruitment, contributing to the heterogeneous MSI-H phenotype. Analysis of variant distribution across additional clinical parameters including age groups, tumor size, differentiation grade, vascular invasion, and distant metastasis is provided in **Supplementary Table S3**.

## 4 Discussion

Our comprehensive analysis of TLR4/MyD88 pathway in colorectal cancer reveals three key findings that advance our understanding of inflammatory signaling in colorectal carcinogenesis. First, we demonstrated distinct expression patterns of TLR4/MyD88 between CRC and adenomas, characterized by significantly higher TLR4 expression in CRC tissues and predominant high-level MyD88 expression in CRC cases. Second, we identified novel genetic variants in both TLR4 and MyD88 genes that significantly influence CRC susceptibility and progression. Third, we established strong associations between TLR4/MyD88 pathway activation and aggressive tumor features, including perineural invasion, high-grade tumor budding, and lymph node metastasis. The detailed examination of these findings and their relationship to current understanding of colorectal cancer biology will be discussed in the following sections.

### 4.1 Expression patterns and their significance

Our study revealed distinct expression patterns of TLR4/MyD88 in colorectal cancer compared to adenomas, with several important implications for understanding CRC pathogenesis. The most striking finding was the significantly higher TLR4 expression in CRC tissues (66.5% vs 30.5%, p<0.001), while MyD88 showed widespread expression in both groups with comparable overall positivity rates (CRC: 97.2%, adenoma: 95.4%). These findings align with current understanding of TLR4/MyD88 signaling in intestinal homeostasis and inflammation (19, 28), where controlled cytokine release and danger signals regulate immune responses while maintaining tolerance to beneficial bacteria.

While TLR4 expression was elevated in CRC compared to adenomas, the majority (61.4%) of TLR4-positive cases showed low expression intensity, particularly in advanced stages. This observation aligns with both Crame et al.’s findings (29) and recent mechanistic studies (30, 31) demonstrating dynamic regulation of TLR4 signaling during tumor progression. This stage-dependent expression pattern supports the evolving concept of inflammation-driven carcinogenesis (16).

The concurrent evaluation of TLR4 and MyD88 expression revealed significant positive correlations (Pearson r=0.372, p<0.001), indicating coordinated pathway activation. This finding supports previous mechanistic studies showing TLR4/MyD88 signaling functions as an integrated pathway in colorectal carcinogenesis (8, 32). Recent molecular analyses have further elucidated how this coordinated signaling influences tumor microenvironment through multiple mechanisms, including NF-κB activation and cytokine production (33, 34). The concurrent evaluation of TLR4 and MyD88 expression revealed significant positive correlations, indicating coordinated pathway activation potentially amplifying downstream oncogenic signals through NF-κB-mediated cytokine production and immune modulation.

In the context of tumor immunity, our findings of predominantly low TLR4 expression in advanced stages could be explained by emerging insights into immune checkpoint regulation. Studies have shown that TLR4 signaling can influence PD-L1 expression and T-cell responses (35, 36), particularly relevant given recent evidence linking innate immune signaling to tumor immune evasion strategies (37, 38).

### 4.2 Molecular and functional implications

The molecular analysis of TLR4/MyD88 signaling in our study revealed complex interactions with implications for CRC progression. The significant correlation between TLR4 and MyD88 expression (Pearson r=0.372, Spearman r=0.373, p<0.001) suggests coordinated pathway activation, potentially amplifying downstream oncogenic signals. High MyD88 expression significantly associated with elevated TLR4 levels (p=0.001), indicating synergistic pathway activation, consistent with the canonical TLR4 signaling cascade (11, 39).

Our findings demonstrate that enhanced TLR4/MyD88 signaling significantly correlates with aggressive tumor features, particularly perineural invasion (p=0.029) and tumor budding (p=0.022). This association aligns with current understanding of pathway-mediated tumor progression through multiple mechanisms. First, TLR4 activation enhances β1 integrin function via AKT phosphorylation, promoting cell adhesion and metastatic potential (40, 41). Second, TLR4/MyD88 signaling activates the urokinase plasminogen activator system through NF-κB, facilitating tumor invasion and metastasis (34, 42).

The pathway’s influence on tumor microenvironment is particularly noteworthy. Combined high TLR4/MyD88 expression significantly associated with lymph node metastasis (p=0.046) and high-grade tumor budding (p=0.002). Recent studies have shown that TLR4/MyD88-mediated COX-2/PGE2 signaling in tumor stromal cells promotes cancer progression through multiple mechanisms: inhibiting apoptosis, enhancing angiogenesis, and modulating immune function (37, 43). This is supported by experimental models showing that MyD88-dependent signaling influences tumor-associated inflammation and progression (44, 45).

### 4.3 Novel genetic variants and their association with CRC risk

Our comprehensive genetic analysis revealed novel variants in both TLR4 and MyD88 genes that significantly influence CRC susceptibility and progression. The TLR4 9:117713042 variant emerged as a significant risk factor, with the AG genotype associated with increased CRC susceptibility (OR=8.92, 95% CI: 1.14-69.95). The predicted damaging effect of this asparagine to serine substitution (PolyPhen-2 score: 0.888) suggests functional implications for TLR4 signaling. Unlike previously reported TLR4 variants in European populations (15, 18), our newly identified variant appears to enhance rather than diminish signaling activity, particularly relevant given recent structural studies of TLR4 domains (46). The exclusive heterozygous presentation of variants in our study reflects their rarity in the Vietnamese population. Notably, the TLR4 9:117713042 variant represents a novel finding in colorectal cancer, located near the well-studied rs4986790 (Asp299Gly) polymorphism on exon 3, suggesting potential functional significance that warrants further investigation.

The MyD88 rs138284536 variant demonstrated even stronger disease association (OR=20.01, 95% CI: 4.72-84.83) and revealed complex interactions with tumor biology. Its association with MSI-H status (45.0% vs 21.5%, p=0.006) yet concurrent low TILs presents an intriguing paradox in MSI-H tumor phenotype (20). This observation can be explained by recent molecular studies showing MyD88-dependent signaling’s dual influence on DNA repair mechanisms and immune cell recruitment (47, 48). The variant’s impact on tumor progression is further evidenced by its associations with mucinous histology (43.5% vs 22.7%, p=0.036), advanced pT stage (29.6% vs 13.2%, p=0.044), and perineural invasion (61.5% vs 22.1%, p=0.004).

In silico analyses predict that both variants could affect protein structure and function, supported by our observation of differential protein expression patterns in variant carriers (49, 50). These structural modifications may explain the enhanced signaling activity suggested by our clinicopathological correlations. The interaction between these genetic variants and the tumor microenvironment appears to be bidirectional, influencing both tumor cells and stromal components (51, 52).

### 4.4 Clinicopathological correlations and prognostic implications

The complex interactions between TLR4/MyD88 signaling and tumor progression revealed in our study provide important insights into CRC biology and potential prognostic stratification. A particularly significant finding was the relationship between TLR4/MyD88 activation and patterns of tumor invasion. The association with perineural invasion suggests involvement in neurotropic spread, a feature increasingly recognized as an independent prognostic factor in CRC (53, 54). This observation aligns with recent mechanistic studies demonstrating that TLR4 activation enhances neural-tumor interactions through NGF/TrkA signaling and chemokine production (55, 56).

The pathway’s influence extends to tumor dissemination, with combined high TLR4/MyD88 expression correlating with lymph node metastasis. Recent studies have shown that TLR4/MyD88-mediated inflammation can modify the lymph node microenvironment through enhanced vascular remodeling (57, 58) and immune modulation (59). Our observation of strong associations with tumor budding carries particular significance, given its emergence as a key prognostic indicator in CRC (60). The relationship between inflammatory signaling and tumor budding provides new insights into epithelial-mesenchymal transition programs crucial for tumor progression (61, 62).

The association between pathway activation and specific tumor subtypes, particularly mucinous histology and MSI status, indicates potential utility in molecular stratification. These relationships acquire greater significance considering recent evidence that inflammatory signaling patterns can predict therapeutic response, especially to immunotherapy (63, 64). The differential expression patterns across tumor subtypes suggest opportunities for personalized therapeutic approaches (65, 66). This concept is supported by recent pan-cancer analyses showing that inflammatory signaling networks can orchestrate multiple hallmarks of cancer progression (67, 68).

### 4.5 Study limitations

Several key limitations should be considered when interpreting our findings. First, study design and statistical power limitations include the moderate single-center sample size (176 CRC, 131 adenoma cases) which may limit generalizability, particularly for genetic associations with wide confidence intervals. The cross-sectional design prevents definitive causal inference, and the absence of homozygous variant genotypes limited comprehensive genetic model analysis and statistical power for recessive inheritance patterns.

Second, molecular analysis limitations encompass the lack of functional validation assays to confirm predicted biological effects of identified genetic variants. While in silico analyses suggest functional impacts, experimental validation through *in vitro* and *in vivo* studies is required to establish causality and confirm structural predictions.

Third, analytical scope limitations include our focus on selected TLR4 and MyD88 variants, which may not capture the full spectrum of inflammatory pathway alterations in CRC. Future larger, multi-center studies with comprehensive molecular profiling, functional validation experiments, and longitudinal follow-up would help validate and extend our observations (69, 70).

## 5 Conclusions

This study establishes three key findings regarding TLR4/MyD88 pathway alterations in colorectal cancer. First, we demonstrated distinct expression patterns between CRC and adenomas, with significantly higher TLR4 expression and predominant high-level MyD88 expression in CRC tissues. Second, we identified novel genetic variants in both TLR4 and MyD88 genes that significantly influence disease susceptibility and progression. Third, we established strong associations between pathway activation and aggressive tumor features, particularly perineural invasion and high-grade tumor budding.

These molecular signatures enhance our understanding of inflammation-driven colorectal carcinogenesis and show promise for improving disease stratification and prognostication. The identification of novel variants and their associations with specific clinicopathological features provides potential biomarkers for risk assessment and therapeutic targeting. Future studies should focus on functional validation of these variants and evaluation of their utility in personalized treatment approaches.

Our findings suggest potential future research directions for TLR4/MyD88 pathway modulation in colorectal cancer, though extensive functional validation and clinical studies would be required before any therapeutic applications could be considered.

## Data Availability

The data presented in the study are deposited in the Zenodo repository, DOI: 10.5281/zenodo.15805321. The dataset is publicly available at: https://zenodo.org/records/15805321.
