# Supplementary material for: TLR4/MyD88 expression patterns and novel genetic variants: association with aggressive clinicopathological features in colorectal cancer
Source: Front Oncol. 2025 Jul 9;15:1568729. doi: 10.3389/fonc.2025.1568729 (PMC12283328; doi:10.3389/fonc.2025.1568729)
Supplement: Supplementary file 1 [file Table1.docx]

Supplementary Material

# Supplementary Tables

**Table S1. Complete Analysis of TLR4 and MyD88 Expression in Relation to All Clinicopathological Features in CRC (n=176)**

| **Parameters** | **TLR4 Expression** | | | **MyD88 Expression** | | | **Combined Score** | | |
| --- | --- | --- | --- | --- | --- | --- | --- | --- | --- |
|  | Low | High | *p-value* | Low | High | *p-value* | <5 | ≥5 | *p-value* |
| **Demographic Features** |  |  |  |  |  |  |  |  |  |
| Sex |  |  |  |  |  |  |  |  |  |
| Male | 61 (89.7) | 7 (10.3) | 0.300 | 40 (37.7) | 66 (62.3) | 0.522 | 74 (67.3) | 36 (32.7) | 0.622 |
| Female | 47 (95.9) | 2 (4.1) |  | 28 (43.1) | 37 (56.9) |  | 42 (63.6) | 24 (36.4) |  |
| Age (years) |  |  |  |  |  |  |  |  |  |
| <60 | 33 (100) | 0 (0) | 0.074 | 17 (36.2) | 30 (63.8) | 0.795 | 29 (60.4) | 19 (39.6) | 0.423 |
| 60-75 | 48 (87.3) | 7 (12.7) |  | 33 (42.3) | 45 (57.7) |  | 52 (65.0) | 28 (35.0) |  |
| >75 | 27 (93.1) | 2 (6.9) |  | 18 (39.1) | 28 (60.9) |  | 35 (72.9) | 13 (27.1) |  |
| **Primary Tumor Characteristics** |  |  |  |  |  |  |  |  |  |
| Location |  |  |  |  |  |  |  |  |  |
| Right colon | 36 (92.3) | 3 (7.7) | 0.187 | 23 (41.1) | 33 (58.9) | 0.730 | 36 (63.2) | 21 (36.8) | 0.865 |
| Left colon | 57 (95.0) | 3 (5.0) |  | 35 (41.2) | 50 (58.8) |  | 59 (67.0) | 29 (33.0) |  |
| Rectum | 15 (83.3) | 3 (16.7) |  | 10 (33.3) | 20 (66.7) |  | 21 (67.7) | 10 (32.3) |  |
| Size |  |  |  |  |  |  |  |  |  |
| <5cm | 71 (94.7) | 4 (5.3) | 0.279 | 38 (36.5) | 66 (63.5) | 0.283 | 66 (61.7) | 41 (38.3) | 0.141 |
| ≥5cm | 37 (88.1) | 5 (11.9) |  | 30 (44.8) | 37 (55.2) |  | 50 (72.5) | 19 (27.5) |  |
| **Histopathological Features** |  |  |  |  |  |  |  |  |  |
| Histological type |  |  |  |  |  |  |  |  |  |
| Conventional | 93 (92.1) | 8 (7.9) | 1.000 | 60 (41.4) | 85 (58.6) | 0.257 | 97 (65.1) | 52 (34.9) | 0.778 |
| Mucinous | 14 (93.3) | 1 (6.7) |  | 7 (29.2) | 17 (70.8) |  | 17 (68.0) | 8 (32.0) |  |
| Differentiation grade |  |  |  |  |  |  |  |  |  |
| Well | 8 (100) | 0 (0) | 1.000 | 4 (36.4) | 7 (63.6) | 0.818 | 7 (63.6) | 4 (36.4) | 0.847 |
| Moderate | 80 (90.0) | 8 (9.1) |  | 53 (41.4) | 75 (58.6) |  | 85 (64.9) | 46 (35.1) |  |
| Poor | 20 (95.2) | 1 (4.8) |  | 11 (34.4) | 21 (65.6) |  | 24 (70.6) | 10 (29.4) |  |
| Histological grade |  |  |  |  |  |  |  |  |  |
| Low | 8 (100) | 0 (0) | 0.516 | 4 (36.4) | 7 (63.6) | 1.000 | 7 (63.6) | 4 (36.4) | 1.000 |
| High | 100 (91.7) | 9 (8.3) |  | 64 (40.0) | 96 (60.0) |  | 109 (66.1) | 56 (33.9) |  |
| **Tumor Progression Features** |  |  |  |  |  |  |  |  |  |
| Distant metastasis |  |  |  |  |  |  |  |  |  |
| Absent | 100 (92.6) | 8 (7.4) | 0.526 | 63 (40.1) | 94 (59.9) | 0.746 | 106 (65.4) | 56 (34.6) | 0.774 |
| Present | 8 (88.9) | 1 (11.1) |  | 5 (35.7) | 9 (64.3) |  | 10 (71.4) | 4 (28.6) |  |
| **Tumor Microenvironment** |  |  |  |  |  |  |  |  |  |
| TILs |  |  |  |  |  |  |  |  |  |
| High | 6 (100) | 0 (0) | 1.000 | 3 (30.0) | 7 (70.0) | 0.396 | 6 (60.0) | 4 (40.0) | 0.447 |
| Moderate | 28 (93.3) | 2 (6.7) |  | 19 (48.7) | 20 (51.3) |  | 31 (73.8) | 11 (26.2) |  |
| Low | 74 (91.4) | 7 (8.6) |  | 46 (37.7) | 76 (62.3) |  | 79 (63.7) | 45 (36.3) |  |
| MSI status |  |  |  |  |  |  |  |  |  |
| MSS | 64 (90.1) | 7 (9.9) | 0.717 | 41 (39.4) | 63 (60.6) | 0.990 | 70 (66.0) | 36 (34.0) | 0.851 |
| MSI-H | 30 (93.8) | 2 (6.2) |  | 17 (39.5) | 26 (60.5) |  | 29 (64.4) | 16 (35.6) |  |

*Data presented as number (percentage); Statistical analysis was performed using Chi-square test or Fisher's exact test where appropriate; No statistically significant associations were observed in this comprehensive analysis (all p-values >0.05); TILs: Tumor-infiltrating lymphocytes; MSS: Microsatellite stable; MSI-H: High-level microsatellite instability; CRC: Colorectal cancer.*

**Table S2. Extended Genetic Model Analysis of TLR4 and MyD88 Variants**

| **Gene/Position** | **Genetic Model** | **Adenoma** | **CRC** | **OR (95% CI)** | ***p*** |
| --- | --- | --- | --- | --- | --- |
| **TLR4** |  | n=85 | n=125 |  |  |
| 9:117713028 | **Dominant** |  |  |  |  |
|  | TT | 82 (96.47) | 117 (93.6) | 1.00 |  |
|  | TG+GG | 3 (3.53) | 8 (6.4) | 1.87 (0.48-7.26) | 0.366 |
|  | **Recessive** |  |  |  |  |
|  | TT+TG | 85 (100) | 125 (100) | 1.00 |  |
|  | GG | 0 (0) | 0 (0) | - | - |
|  | **Alleles** |  |  |  |  |
|  | T | 167 (98.24) | 242 (96.8) | 1.00 |  |
|  | G | 3 (1.76) | 8 (3.2) | 1.84 (0.48-7.04) | 0.373 |
| rs1444566743 | **Dominant** |  |  |  |  |
|  | AA | 78 (91.76) | 113 (90.4) | 1.00 |  |
|  | AG+GG | 7 (8.24) | 12 (9.6) | 1.18 (0.45-3.14) | 0.735 |
|  | **Recessive** |  |  |  |  |
|  | AA+AG | 85 (100) | 125 (100) | 1.00 |  |
|  | GG | 0 (0) | 0 (0) | - | - |
|  | **Alleles** |  |  |  |  |
|  | A | 163 (95.88) | 238 (95.2) | 1.00 |  |
|  | G | 7 (4.12) | 12 (4.8) | 1.17 (0.45-3.05) | 0.741 |
| 9:117713042 | **Dominant** |  |  |  |  |
|  | AA | 84 (98.82) | 113 (90.4) | 1.00 |  |
|  | AG+GG | 1 (1.18) | 12 (9.6) | 8.92 (1.14-69.95) | 0.037* |
|  | **Recessive** |  |  |  |  |
|  | AA+AG | 85 (100) | 125 (100) | 1.00 |  |
|  | GG | 0 (0) | 0 (0) | - | - |
|  | **Alleles** |  |  |  |  |
|  | A | 169 (99.41) | 238 (95.2) | 1.00 |  |
|  | G | 1 (0.59) | 12 (4.8) | 8.52 (1.10-66.16) | 0.041* |
| **MyD88** |  | **n=119** | **n=153** |  |  |
| rs2125780689 | **Dominant** |  |  |  |  |
|  | GG | 102 (85.71) | 150 (98.04) | 1.00 |  |
|  | GA+AA | 17 (14.29) | 3 (1.96) | 0.12 (0.03-0.42) | <0.001* |
|  | **Recessive** |  |  |  |  |
|  | GG+GA | 119 (100) | 153 (100) | 1.00 |  |
|  | AA | 0 (0) | 0 (0) | - | - |
|  | **Alleles** |  |  |  |  |
|  | G | 221 (92.86) | 303 (99.02) | 1.00 |  |
|  | A | 17 (7.14) | 3 (0.98) | 0.13 (0.04-0.44) | 0.001* |
| rs138284536 | **Dominant** |  |  |  |  |
|  | CC | 117 (98.32) | 114 (74.51) | 1.00 |  |
|  | CA+AA | 2 (1.68) | 39 (25.49) | 20.01 (4.72-84.83) | <0.001* |
|  | **Recessive** |  |  |  |  |
|  | CC+CA | 119 (100) | 153 (100) | 1.00 |  |
|  | AA | 0 (0) | 0 (0) | - | - |
|  | **Alleles** |  |  |  |  |
|  | C | 236 (99.16) | 267 (87.25) | 1.00 |  |
|  | A | 2 (0.84) | 39 (12.75) | 17.24 (4.12-72.15) | <0.001* |

*Data presented as number (percentage); Adenoma group: n=85 for TLR4, n=119 for MyD88; Colorectal cancer (CRC) group: n=125 for TLR4, n=153 for MyD88; OR: Odds ratio; CI: Confidence interval; ORs were calculated using logistic regression analysis; *Statistically significant (p<0.05).*

**Table S3. Additional Clinicopathological Features in Relation to TLR4 and MyD88 Variants**

| **Features** | **TLR4 9:117713042** | | | **MyD88 rs138284536** | | |
| --- | --- | --- | --- | --- | --- | --- |
|  | Wild-type | Variant | *p* | Wild-type | Variant | *p* |
| **Clinical Features** |  |  |  |  |  |  |
| Age (years) |  |  |  |  |  |  |
| <60 | 35 (89.7) | 40 (10.3) | 0.929 | 31 (73.8) | 11 (26.2) | 0.768 |
| 60-75 | 48 (87.3) | 7 (12.7) |  | 50 (72.5) | 19 (27.5) |  |
| >75 | 27 (93.1) | 2 (6.9) |  | 33 (78.6) | 9 (21.4) |  |
| **Tumor location** |  |  |  |  |  |  |
| Right colon | 32 (86.5) | 5 (13.5) | 0.336 | 37 (72.5) | 14 (27.5) | 0.694 |
| Left colon | 81 (92.0) | 7 (8.0) |  | 77 (75.5) | 25 (24.5) |  |
| **Tumor Characteristics** |  |  |  |  |  |  |
| Size |  |  |  |  |  |  |
| <5cm | 74 (90.2) | 8 (9.8) | 0.604 | 74 (79.6) | 19 (20.4) | 0.074 |
| ≥5cm | 39 (90.7) | 4 (9.3) |  | 40 (66.7) | 20 (33.3) |  |
| Differentiation grade |  |  |  |  |  |  |
| Well/Moderate | 95 (92.2) | 8 (7.8) | 0.222 | 95 (77.9) | 27 (22.1) | 0.059 |
| Poor | 18 (81.8) | 4 (18.2) |  | 19 (61.3) | 12 (38.7) |  |
| **Metastatic Status** |  |  |  |  |  |  |
| Lymph node metastasis |  |  |  |  |  |  |
| Absent | 62 (88.6) | 8 (11.4) | 0.434 | 67 (76.1) | 21 (23.9) | 0.591 |
| Present | 51 (92.7) | 4 (7.3) |  | 47 (72.3) | 18 (27.7) |  |
| Distant metastasis |  |  |  |  |  |  |
| Absent | 106 (90.6) | 11 (9.4) | 0.565 | 106 (75.7) | 34 (24.3) | 0.318 |
| Present | 7 (87.5) | 1 (12.5) |  | 8 (61.5) | 5 (38.5) |  |
| Vascular invasion |  |  |  |  |  |  |
| Absent | 96 (88.9) | 12 (11.1) | 0.368 | 104 (73.3) | 36 (25.7) | 0.567 |
| Present | 17 (100) | 0 (0) |  | 10 (76.9) | 3 (23.1) |  |

*Data presented as number (percentage); Wild-type: AA for TLR4, CC for MyD88; Variant: AG for TLR4, CA for MyD88 Statistical analysis performed using Chi-square test or Fisher's exact test where appropriate; All features analyzed in CRC patients only.*
